# The 8q24 region hosts miRNAs altered in biospecimens of colorectal and bladder cancer patients

**DOI:** 10.1002/cam4.5375

**Published:** 2022-11-10

**Authors:** Amedeo Gagliardi, Giulia Francescato, Giulio Ferrero, Giovanni Birolo, Sonia Tarallo, Antonio Francavilla, Giulia Piaggeschi, Carla Di Battista, Gaetano Gallo, Alberto Realis Luc, Carlotta Sacerdote, Giuseppe Matullo, Paolo Vineis, Alessio Naccarati, Barbara Pardini

**Affiliations:** ^1^ Italian Institute for Genomic Medicine (IIGM), c/o IRCCS Candiolo Turin Italy; ^2^ Department of Computer Science University of Turin Turin Italy; ^3^ Department of Clinical and Biological Sciences University of Turin Turin Italy; ^4^ Department of Medical Sciences University of Turin Turin Italy; ^5^ Candiolo Cancer Institute, FPO‐IRCCS Candiolo, Turin Italy; ^6^ Universidad Católica San Antonio de Murcia (UCAM) Campus de los Jerónimos Guadalupe, Murcia Spain; ^7^ Department of Colorectal Surgery Clinica S. Rita Vercelli Italy; ^8^ Department of Surgical Science Sapienza University of Rome Rome Italy; ^9^ Unit of Cancer Epidemiology, Center for Cancer Prevention (CPO‐Piemonte) Turin Italy; ^10^ MRC Center for Environment and Health, Imperial College London UK

**Keywords:** 8q24 genomic region, bladder cancer, colorectal cancer, microRNAs, stool, urine

## Abstract

**Background:**

The 8q24 locus is enriched in cancer‐associated polymorphisms and, despite containing relatively few protein‐coding genes, it hosts the *MYC* oncogene and other genetic elements connected to tumorigenesis, including microRNAs (miRNAs). Research on miRNAs may provide insights into the transcriptomic regulation of this multiple cancer‐associated region.

**Material and methods:**

We profiled all miRNAs located in the 8q24 region in 120 colorectal cancer (CRC) patients and 80 controls. miRNA profiling was performed on cancer/non‐malignant adjacent mucosa, stool, and plasma extracellular vesicles (EVs), and the results validated with The Cancer Genome Atlas (TCGA) data. To verify if the 8q24‐annotated miRNAs altered in CRC were dysregulated in other cancers and biofluids, we evaluated their levels in bladder cancer (BC) cases from the TCGA dataset and in urine and plasma EVs from a set of BC cases and healthy controls.

**Results:**

Among the detected mature miRNAs in the region, 12 were altered between CRC and adjacent mucosa (adj. *p* < 0.05). Five and four miRNAs were confirmed as dysregulated in the CRC and BC TCGA dataset, respectively. A co‐expression analysis of tumor/adjacent tissue data from the CRC group revealed a correlation between the dysregulated miRNAs and CRC‐related genes (*PVT1* and *MYC*) annotated in 8q24 region. miR‐30d‐5p and miR‐151a‐3p, altered in CRC tissue, were also dysregulated in stool of CRC patients and urine of BC cases, respectively. Functional enrichment of dysregulated miRNA target genes highlighted terms related to TP53‐mediated cell cycle control.

**Conclusions:**

Altered expression of 8q24‐annotated miRNAs may be relevant for the initiation and/or progression of cancer.

## Graphical abstract

The work provided new insights into the transcriptomic regulation of the 8q24 locus, a region enriched in multiple cancer‐associated polymorphisms. Among the 54 mature miRNAs in the region, 12 were altered in colorectal cancer tissues with 5 miRNAs validated in TCGA dataset. miR‐30d‐5p and miR‐151a‐3p resulted also dysregulated in stool of CRC patients and urine of bladder cancer cases, highlighting the importance of 8q24‐annotated miRNAs for the initiation and/or progression of cancer.

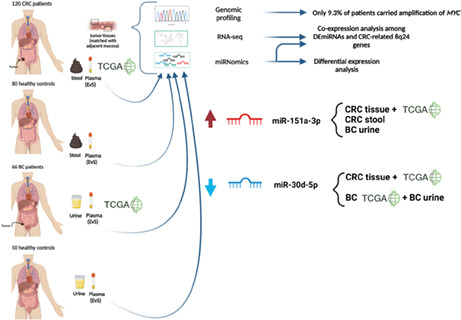

## 1 INTRODUCTION

In the past, specific genomic regions have been strongly associated with increased susceptibility to different cancers.^1^ An example is represented by a quite large region (>4 Mb) mapping to the human chromosome 8q24 (chr.8q24).^2^ Several genome wide association studies (GWAS) have identified in this locus a considerable number of genetic variants linked to predisposition to different cancers, including prostate, breast, esophagus, head and neck, ovarian, colon, bladder, and pancreas.^3^, ^4^ However, the locus has been described for years as a “gene desert” due to the relatively low number of protein coding genes mapped in the region.^2^

Together with the associations observed by GWAS, the 8q24 locus presents some relevant features for oncogenesis that have captured the attention of researchers. Indeed, *MYC* oncogene, which resides in 8q24, could represent an ideal candidate to functionally explain the observed cancer risk associations. This gene is the most frequently amplified protein coding gene across cancers and is involved in 20% of human cancers.^5^, ^6^ *MYC* is a fundamental transcription factor regulating cell cycle, metabolism, ribosome biogenesis and cell adhesion.^6^ Several cancer‐associated genetic polymorphisms are located within the surrounding non‐coding region of *MYC*, implicating that these variants may alter regulatory elements of *MYC* expression.^3^ However, until now, no direct correlation between *MYC* transcriptional activities and the cancer‐associated variability of the region has been demonstrated.^2^, ^7^

The same locus hosts other genetic elements potentially interesting, such as genes (e.g., *FAM84B*, *GSDMC*, *FAM49B*, and *ASAP1*) that have been connected to tumorigenesis^8^, ^9^, ^10^, ^11^ but also important pseudogenes and other complex transcriptional regulatory components. For example, *POU5F1B,* a pseudogene of the stem cell self‐renewal gene *OCT4*, is frequently amplified in cancer and has been shown to promote colony formation, tumorigenicity, tumor growth, angiogenesis, and cell proliferation.^12^ A considerable number of cancer‐connected long non‐coding RNAs (*PCAT1*, *CASC19*, *PRNCR1*, *CCAT1*, *CASC8*, *CCAT2* and *CASC11*, upstream of *MYC*; and *PVT1*, *LINC00924*, and *CCDC26*, downstream of *MYC*) have been identified in 8q24 and aroused the interest of researchers including us.^13^, ^14^ Interestingly, this region hosts a large number of other small non‐coding RNAs, including 30 genes encoding for 54 mature microRNAs (miRNAs) (mirBase release v.22.1^15^) (Figure S1), such as the miR‐1204‐1208a miRNA cluster^16^ in the *PVT1* gene.

Despite all the known genetic features, a deep characterization of miRNA profiles in the 8q24 region in association with cancer is still missing. In this respect, altered levels of miRNAs may provide further insights into the processes that involve this key genomic locus in cancer.

In the present study, we focused on all miRNAs residing in 8q24 and evaluated their expression levels in both tumor tissue and non‐malignant adjacent mucosa of colorectal cancer (CRC) patients from the whole miRNome analysis. miRNA expression dysregulation in the 8q24 region was also validated in The Cancer Genome Atlas (TCGA) database and further tested in non‐invasive biofluids such as stool and plasma extracellular vesicles (EVs) from the same CRC patients and additional healthy controls at colonoscopy. To assess if those miRNA alterations also occur in other cancers, we tested the levels of such miRNAs in the bladder cancer (BC) dataset of TCGA database. We further verified the obtained results in urine and plasma EVs samples from an independent set of BC patients and controls addressing the functional role of the genes targeted by the dysregulated miRNAs. We found a high dysregulation of miR‐30d‐5p and miR‐151a‐3p in tissues that had a reflection also on biofluids.

## 2 MATERIALS AND METHODS

### 2.1 Study population

#### 2.1.1 Study I

The Study I population consisted of 200 subjects (89 women and 111 men) recruited at the Clinica S. Rita of Vercelli. Based on the results of the colonoscopy, participants were categorized as healthy controls (80 subjects with negative colonoscopy for tumor or other gastrointestinal disorders) or CRC patients (120 subjects).^17^ CRC patients were also stratified according to the localization of the tumor (colon/sigma‐region or rectum), the stage of the cancer (stage 0–IV)^18^ and its grade (G1–G3).^18^ Stool and blood samples were collected at the time of recruitment and, for CRC cases only, tissue pairs of primary tumor and adjacent non‐malignant mucosa were also collected at the surgery.

All recruited subjects signed a written consent to participate in the study, in agreement with the Helsinki Declaration. The design of the study, informed consent and protocols were approved by the local Ethics Committees (Azienda Ospedaliera “SS. Antonio e Biagio e C. Arrigo” of Alessandria. ID: Colorectal miRNA CEC2014).

#### 2.1.2 Study II

The Study II consisted of a set of subjects recruited in the context of previous research^19^ nested in the Turin Bladder Cancer Study^20^, ^21^ and consisting of 116 men. Sixty‐six men were diagnosed with BC and the remaining 50 subjects were age‐matched healthy controls. Among the 66 men with BC, 10 resulted muscle invasive (MIBC) and 56 non‐muscle invasive (NMIBC). Patients were newly diagnosed with a confirmed histology of BC. Controls were males randomly recruited among patients treated for non‐neoplastic disease in the same urology departments of the cases. All patients presenting other cancers, liver, or renal diseases as well as smoking‐related conditions were excluded. A complete description of the Study II population is available elsewhere.^19^, ^22^

For all the subjects, urine and plasma samples were collected. Subjects signed a written consent to participate in the study in agreement with the Helsinki Declaration. The design of the study, informed consent, and protocols were approved by the local Ethics Committees (Interhospital Ethical Board of San Giovanni Battista/C.T.O./C.R.F./Maria Adelaide hospitals, Turin, and the Institutional Review Board of the Italian Institute for Genomic Medicine (IIGM)).

### 2.2 Sample collection

#### 2.2.1 Tissues

Primary tumor and adjacent mucosa (at least 20 cm distant) tissues from CRC subjects (Study I) were collected during surgical resection and immediately transferred in cryogenic vials with RNAlater™ Solution (Invitrogen) and stored at −80°C until use.

#### 2.2.2 Stool

Fecal samples were obtained from all patients of Study I that were instructed to self‐collect the specimen at home in stool nucleic acid collection and transport tubes with RNA stabilizing solution (Norgen Biotek Corp.). Aliquots (200 μl) were stored at −80°C until RNA extraction. Further details are described in.^17^

#### 2.2.3 Urine

Urine samples were collected in the morning from all the participants in the Study II and stored at 4°C until they were centrifuged at 3000*g* for 10 min. The urine supernatant aliquots were then transferred in tubes and stored at −80°C until use.

#### 2.2.4 Plasma

For both study cohorts, plasma samples were obtained from 5–8 ml of blood collected in EDTA tubes, centrifuged at 1000 rpm for 10 minutes. From each tube of blood, about 1–2 ml of plasma was obtained and distributed in 200 μl aliquots stored at −80°C until use. EVs were isolated from 200 μl of plasma using the ExoQuick™ Exosome Precipitation Solution (System Biosciences, Mountain View, CA, USA) as in.^23^ Briefly, plasma was mixed with 50.4 μl of ExoQuick™ Solution and refrigerated at 4°C overnight (at least 12 h). The mixture was then centrifuged at 1500 g for 30 min. EVs pellets were dissolved in 200 μl of nuclease‐free water and RNA was extracted immediately from this solution. Further details are described in.^23^

### 2.3 RNA extraction

Extraction of total RNA from stool, urine, plasma EVs, and tissues was performed using appropriate kits/methodologies for total RNA purification according to the specimen to be analyzed.

RNA from tissues was isolated using QIAzol (QIAGEN) after tissue homogenization performed with ULTRA‐TURRAX® Homogenizer,^13^ followed by phenol/chloroform extraction according to the manufacturer's standard protocol.

Total RNA from stool samples was extracted with the Stool Total RNA Purification Kit (Norgen Biotek Corp.) according to the manufacturer's protocol. Total RNA from plasma EVs was extracted with the miRNeasy Plasma/Serum Mini‐kit (QIAGEN) using the QIAcube extractor (QIAGEN). Total RNA from urine samples was extracted with the Urine microRNA Purification Kit (Norgen Biotek Corp.), following the manufacturer's standard protocol. The RNA concentration was quantified by Qubit™ 4 fluorometer with Qubit™ microRNA or RNA Broad range Assay kits (Invitrogen).

### 2.4 Library preparation for small RNA sequencing (small RNA‐seq)

Small RNA‐seq libraries were prepared from RNA extracted from tissues, stool, plasma EVs, and urine. The NEBNext® Multiplex Small RNA Library Prep for Illumina® (New England Biolabs, Inc.) kit was used to convert small RNA transcripts into barcoded cDNA libraries. For each library, 6 μl of RNA (35 ng for EVs RNA and 250 ng for tissue/stool/urine RNA) were processed as starting material. Each library was prepared with a unique indexed primer. Multiplex adapter ligations, reverse transcription primer hybridization, reverse transcription reaction and PCR amplification were performed according to the manufacturer's protocol. After PCR amplification, the cDNA constructs were purified with the QIAQuick® PCR Purification Kit (QIAGEN), following the modifications suggested by the NEBNext® Multiplex Small RNA Library Prep for Illumina® protocol. Final libraries were loaded on the Bioanalyzer® 2100 (Agilent Technologies) using the DNA High Sensitivity Kit (Agilent Technologies) according to the manufacturer's protocol. Libraries were pooled together (in 24‐plex) and further purified with a gel size selection. A final Bioanalyzer® 2100 run with the High Sensitivity DNA Kit (Agilent Technologies) allowed to assess DNA libraries quality regarding size, purity, and concentration.^24^ The obtained libraries were subjected to the Illumina® sequencing pipeline, passing through clonal cluster generation on a single‐read flow cell (Illumina) by bridge amplification on the cBot (TruSeq SR Cluster Kit v3‐cBOT‐HS, Illumina) and 50 cycles sequencing‐by‐synthesis on the HiSeq™ 2000 Sequencing System (Illumina) (in collaboration with EMBL, Gene core facility).

### 2.5 Library preparation for total RNA sequencing (RNA‐seq)

Before RNA‐seq library preparation, total RNA from tissue samples was cleaned up and DNase‐treated with the RNA Clean & Concentrator™‐5 kit, following manufacturer's protocol (Zymo Research) to remove all traces of DNA. Next, the quality of the input RNA was determined by RNA Integrity Number (RIN) measurement obtained by running the samples on an Agilent Bioanalyzer® RNA 6000 Nano chip (Agilent Technologies). For each sample, 500 ng of RNA was used as starting material to libraries preparation. RNA‐seq libraries were prepared with the NEBNext® Ultra II Directional RNA Library Prep for Illumina® kits (New England Biolabs) after ribosomal RNA depletion, following manufacturer's instructions. The generated barcoded libraries of about 300 bp fragments were run on an Illumina® NovaSeq™ 6000 platform (Illumina).

### 2.6 DNA extraction

For DNA extraction, tissues were initially homogenized in a homogenization solution (Promega) and then processed with Maxwell® RSC Tissue DNA Kit (Promega). Before loading samples onto Maxwell® RSC Cartridges, 300 μl of Lysis Buffer and 30 μl of Proteinase K were added to the homogenized samples, and further incubated for 20 min at 56°C. DNA was quantified with Qubit™ 4 fluorometer using Qubit™ dsDNA High Sensitivity Assay Kit (Invitrogen).

### 2.7 TruSight™ oncology 500 high‐throughput

DNA libraries were prepared using the hybrid capture based TruSight™ Oncology 500 High Throughput (TSO500‐HT) Library Preparation Kit (Illumina) following Illumina® TruSight™ Oncology 500 Reference Guide (document # 1000000094853 v02). In brief, the genomic DNA was fragmented using the Covaris® ME220 focused‐ultrasonicator (Covaris) for 10 seconds at 50 W. After end repair, A‐tailing, and adapter ligation, the adapter‐ligated fragments were amplified using primers to add index sequences for sample multiplexing. Libraries were enriched through two hybridization/capture steps using specific probes: a pool of oligos specific to 523 genes targeted by TSO500‐HT was hybridized to the DNA libraries overnight. Then, streptavidin magnetic beads were employed to capture probes hybridized to the targeted regions. PCR amplification, cleanup, and quantification of the enriched DNA using Qubit™ dsDNA HS Assay Kit (Invitrogen) were the final steps. Following pooling and denaturation, libraries were diluted to the appropriate loading concentration and finally sequenced on Illumina® NovaSeq™ 6000 Sequencer (Illumina) (read length of 200 bp paired end).

### 2.8 Expression profiles of miRNAs in 8q24 in TCGA database

Data from TCGA related to CRC (COAD‐colon adenocarcinoma and READ rectum adenocarcinoma) and BC (BLCA–Bladder carcinoma) were retrieved from the GDC data portal (https://portal.gdc.cancer.gov/; v.29.0). For each project, the *isoform_expression_quantification.txt* file, containing information on miRNA expression levels as raw counts, was downloaded along with information on the coordinates for each miRNA and their accession number on miRBase (v.22.1). Raw counts were then normalized using the DESeq2 package (v.1.28.1)^25^ for the statistical software R (v. 4.0.2). TCGA‐COAD and TCGA‐READ data were merged in the same dataset of CRC patients. Files containing clinical data, information on patient biospecimen, and metadata were downloaded from the TCGA Data portal (v.29.0). To prepare the count matrix, each mature miRNA name was retrieved with the use of the ‘miRBaseConverter’^26^ R package. Data were filtered to keep only white‐Caucasian individuals. Differential expression analyses were performed initially on tumor tissue samples and paired adjacent mucosa and then considering also non‐matched tumor samples.

### 2.9 Computational and statistical analyses

miRNA data analysis was performed as previously described in.^17^, ^23^ FASTQ files from small RNA sequencing were quality‐checked with FASTQC software. All reads shorter than 14 nucleotides were discarded, and the remaining reads were trimmed from the adapters using the CutAdapt software.^27^ The clipped reads were mapped against the precursor miRNA sequences downloaded from miRBase (v.22.1) with the use of BWA algorithm.

miRNA normalization and differential expression analyses (Wald test) were performed with the DESeq2 package.^25^ A miRNA was defined as differentially expressed (DE) with a Benjamini–Hochberg‐adjusted p‐value lower than 0.05. The differential expression analysis was initially performed comparing cancer cases/tissues versus controls/adjacent mucosa in both cohorts. Then, for Study I subjects, the analysis was also conducted stratifying CRC samples according to the tumor localization (colon/sigma or rectum), stage (from I to IV), and grade (from 1 to 3). All the analyses were adjusted also for sex (not for Study II), age, and library pool.

After performing the differential analyses on all the miRNome, among all the expressed miRNAs, those localized in the 8q24 region were extracted considering their genomic position from miRBase (v.22.1). Specifically, only miRNAs expressed and located in the interval chr8:116,700,636‐145,138,000 (Reference genome GRCh38/hg38) were retained. Among them, only those associated with a median normalized count level >10 were used for the analysis.

To guarantee the reproducibility of the reported results, the raw read count tables and subjects metadata discussed in the present work are reported in https://doi.org/10.6084/m9.figshare.20765929.

RNA‐seq data were pre‐processed using FastP (v.0.21)^28^ with default settings and then aligned on Gencode annotations (v.36) using Salmon tool (v.1.4) with default settings and with *seqBias* and *gcBias* options to correct for hexamer priming and GC biases, respectively. The read counts were then normalized in transcripts per million (TPM) with the txImport R package (v.1.22).^29^ Paired differential expression analysis was performed with DESeq2 package using the Wald test.

CRC relevant genes, annotated in the 8q24 region, were identified from recent literature and a co‐expression analysis between those genes and the levels of differentially expressed miRNAs was performed with Spearman correlation method. A miRNA‐gene pair was considered related if associated with a correlation *p* < 0.05.

Data produced by the TSO500‐HT were analyzed with the Illumina TSO500 Local App (v 2.2) pipeline. Briefly, for each subject, files reporting information on quality controls, genetic variants, and tumor mutational burden were created. An in‐house Python script was created to aggregate these files.

### 2.10 Functional enrichment analysis of miRNA targets

To perform a functional enrichment analysis, miRNA target genes were retrieved using miRWalk database (v 2.0) as previously described in Sabo et al.^22^ Only miRTarBase validated interactions involving miRNAs targeting the gene 3′UTR and associated with a score greater or equal than 0.95 were retained. Target genes were subset based on miRNAs log2 fold‐change (log2FC, up‐ or down‐regulated) and separately analyzed with the Metascape web tool^30^ to retrieve the enriched functional terms.

### 2.11 Co‐expression analysis in TCGA datasets

Gene expression data of TCGA‐CO‐READ and TCGA‐BLCA were downloaded from the GDC data portal using the ‘TCGAbiolinks’ package (v2.24.3) for R. The raw counts were normalized using the DESeq2 package (v. 1.28.1). A co‐expression analysis between dysregulated miRNAs and validated genes targeted by at least two miRNAs was performed using the Spearman correlation method. A correlation was considered significant if associated with an adjusted p < 0.05.

### 2.12 Evaluation of *MYC* amplification in TCGA datasets

Copy number alteration (CNA) data related to colorectal cancer (TCGA‐COAD and READ), bladder cancer (TCGA‐BLCA), ovarian cancer (TCGA‐OV), breast cancer (TCGA‐BRCA), stomach adenocarcinoma (TCGA‐STAD), lung squamous cell carcinoma (TCGA‐LUSC) and prostate adenocarcinoma (TCGA‐PRAD) were downloaded from the ‘cBioPortal for Cancer Genomics’ website using the ‘cBioPortalData’ package (v.2.2.11) for R. The raw data were structured in the GISTIC format,^31^ and *MYC* was considered as amplified with a GISTIC score of +2.

## 3 RESULTS

### 3.1 Study I

#### 3.1.1 Study population

The Study I cohort consisted of 120 CRC patients and 80 controls. The mean age of CRC cases was 70.5 ± 10.5 years, with male (*n* = 71) representing 59.2% of the cases. The control group mean age was 57.9 ± 11.3 years, with 40 males and 40 females (Table 1). Stool samples were available for 62 patients, while plasma EVs were available for 53 cases. For the control group, stool and plasma EVs were available for all individuals. Tissue pairs were available for 108 patients. Forty‐three CRC patients had all three biospecimens collected. In this population, 53 out of the 54 known miRNAs in the 8q24 region were detected in all specimens analyzed (tissue, stool, and plasma EVs samples).

**TABLE 1:** Description of the study populations.

Covariate	Study I		Study II	
	Healthy (*n* = 80)	CRC (*n* = 120)	Healthy (*n* = 50)	BC (*n* = 66)
Age				
Mean (SD)	57.9 (± 11.4)	70.5 (± 10.5)	64.6 (± 7.4)	64.2 (± 6.9)
Median (min, max)	58.0 (39.0, 81.0)	70.0 (48.0, 92.0)	66.0 (46.0, 75.0)	65.0 (45.0, 74.0)
Sex				
M	39 (49.4%)	71 (59.2%)	50 (100.0%)	66 (100.0%)
F	40 (50.6%)	49 (40.8%)	0 (0.0%)	0 (0.0%)
Smoking				
Never	30 (37.5%)	52 (43.3%)	18 (36.0%)	25 (37.9%)
Former	32 (40.0%)	36 (30.0%)	26 (52.0%)	34 (51.5%)
Current	13 (16.25%)	16 (13.3%)	5 (10.0%)	7 (10.6%)
NA	5 (6.25%)	16 (13.3%)	1 (2.0%)	0 (0.0%)
T				
Ta	–	–		29 (43.9%)
T1	–	15 (12.5%)	–	24 (36.4%)
T2	–	20 (16.6%)	–	10 (15.2%)
T3	–	66 (55.0%)	–	–
T4	–	8 (6.7%)	–	–
Tis	–	2 (1.7%)	–	3 (4.5%)
NA	–	9 (7.5%)	–	–
N				
N0	–	61 (50.8%)	–	–
N1	–	24 (20.1%)	–	–
N2	–	19 (15.8%)	–	–
NA	–	16 (13.3%)	–	–
M				
M0	–	102 (85.0%)	–	–
M1	–	8 (6.7%)	–	–
NA	–	10 (8.3%)	–	–
Stage				
0	–	2 (1.7%)	–	–
I–II	–	59 (49.2%)	–	–
III–IV	–	45 (37.4%)	–	–
NA	–	14 (11.7%)	–	–
Grade				
G1–G2	–	63 (52.5%)	–	–
G3	–	43 (35.8%)	–	–
NA	–	14 (11.7%)	–	–
Localisation				
Colon‐Sigma	–	70 (58.3%)	–	–
Rectum	–	44 (36.7%)	–	–
NA	–	6 (5.0%)	–	–
BC Grade (1973)				
G1	–	–	–	14 (21.2%)
G2	–	–	–	25 (37.9%)
G3	–	–	–	27 (40.9%)
BC Grade (2004)				
Low‐grade	–	–	–	25 (36.2%)
High‐grade	–	–	–	44 (63.8%)

Abbreviations: BC, bladder cancer; CRC, colorectal cancer; NA, not available; SD, standard deviation.

#### 3.1.2 Small RNA sequencing results in CRC tissue samples

**FIGURE 1:**
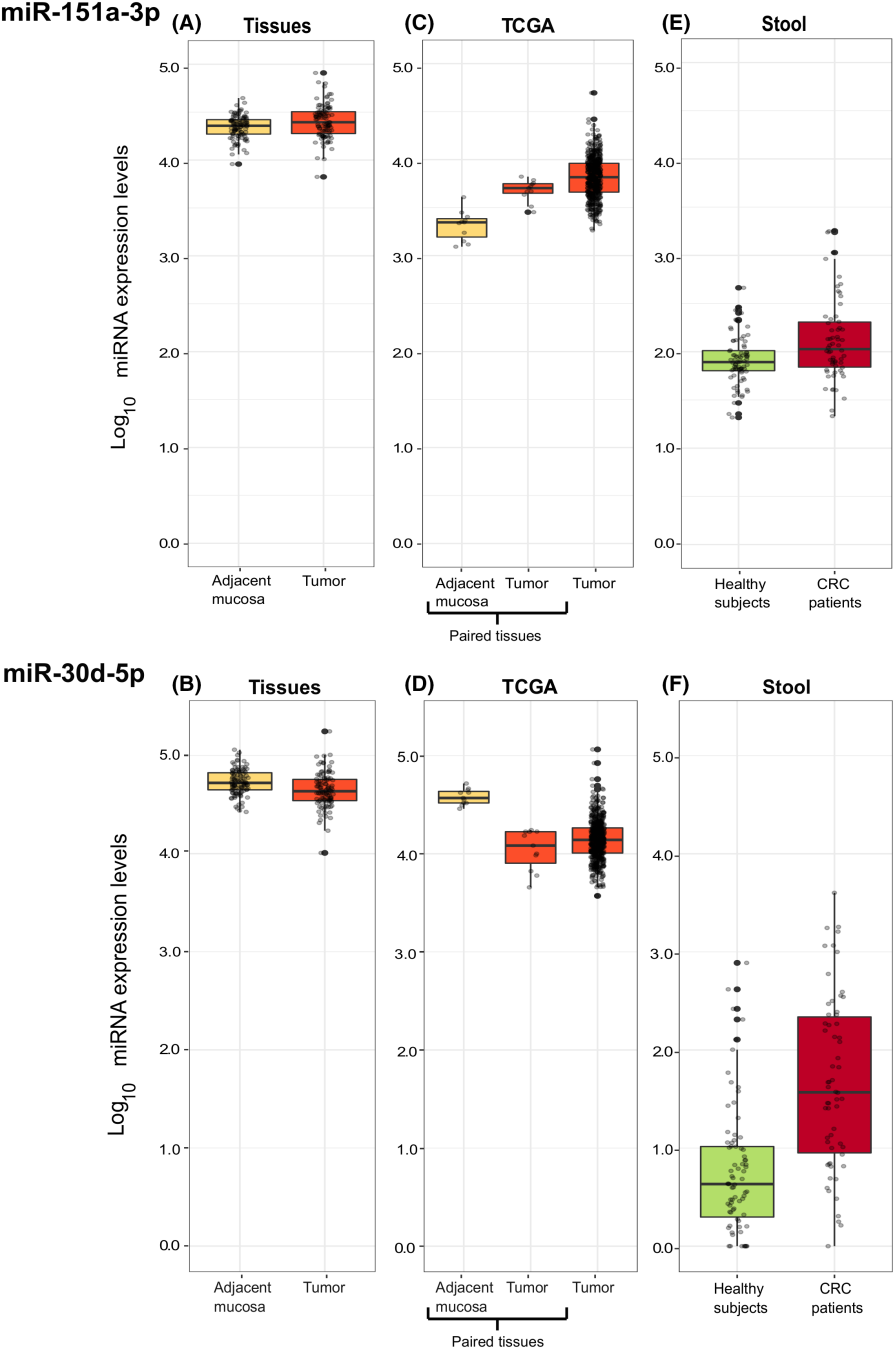
Box plots representing expression levels respectively of miR‐151a‐3p and miR‐30d‐5p in (A, B) CRC tissues versus normal adjacent mucosa from Study I; (C, D) in the TCGA‐CRC database (both for paired and not paired tissues); (E, F) in stool samples from CRC patients and healthy controls of Study I.

Twelve miRNAs were differentially expressed (DE; adj *p* < 0.05) in cancer tissues when compared with the adjacent mucosa. Among them, nine resulted up‐regulated: miR‐151a‐3p (log_2_FC = 0.23; Figure 1A), miR‐151a‐5p (log_2_FC = 0.22), miR‐548az‐5p (log_2_FC = 0.37), miR‐548d‐3p (log_2_FC = 0.38), miR‐937‐3p (log_2_FC = 0.84), miR‐939‐5p (log_2_FC = 0.98), miR‐1302 (log_2_FC = 1.16), miR‐4472 (log_2_FC = 0.32), and miR‐4664‐3p (log_2_FC = 1.76) (Table S1A). Three DE miRNAs were down‐regulated: miR‐30d‐5p (log_2_FC = −0.23; Figure 1B), miR‐30b‐3p (log_2_FC = −0.31), and miR‐4662a‐5p (log_2_FC = −0.46) (Table S1A).

Seven miRNAs (namely miR‐30b‐3p, miR‐30d‐5p, miR‐548d‐3p, miR‐937‐3p, miR‐939‐5p, miR‐1302, miR‐4664‐3p) resulted differentially expressed in both tumors with stages I‐II and III‐IV in comparison to their respective adjacent tissues. Specifically, miR‐30d‐5p also showed a decreasing trend of expression going from normal tissues to stage III‐IV tumors; conversely, miR‐937‐3p and miR‐939‐5p showed an increasing expression trend (Table S1B).

When tumors were stratified for grade, six miRNAs (miR‐151a‐3p, miR‐548d‐3p, miR‐937‐3p, miR‐939‐5p, miR‐1302, and miR‐4664‐3p) were up‐regulated in G1‐G2 cancers vs adjacent mucosa. Conversely, miR‐30b‐3p and miR‐30d‐5p were down‐regulated. In G3 CRC, miR‐548az‐5p, miR‐548d‐5p, miR‐937‐3p, miR‐939‐5p, miR‐1302, miR‐4664‐3p, and miR‐4472 were significantly up‐regulated, while miR‐30b‐5p, miR‐30d‐5p, and miR‐4662a‐5p resulted down‐regulated compared to adjacent tissues (Table S1C).

Finally, miRNA expression was evaluated according to cancer localization. Ten miRNAs were dysregulated in colon/sigma cancer tissues when compared to adjacent mucosa: miR‐151a‐3p, miR‐548az‐5p, miR‐548d‐3p, miR‐937‐3p, miR‐939‐5p, miR‐1302, and miR‐4664‐3p were up‐regulated while miR‐30b‐3p, miR‐30d‐5p, and miR‐4662a‐5p were down‐regulated. Six of these 10 miRNAs were also differentially expressed in rectal cancers with the same expression trend (Table S1D).

#### 3.1.3 Validation on CRC tissues from TCGA

8q24‐annotated miRNAs were investigated on the TCGA dataset. Out of the 52 retrievable miRNAs, four were up‐regulated in tumor tissues with respect to normal adjacent mucosa (22 matched samples): miR‐151a‐3p (log_2_FC = 1.82 Figure 1C), miR‐151a‐5p (log_2_FC = 0.84), miR‐30b‐5p (log_2_FC = 2.84), and miR‐4662a‐5p (log_2_FC = 3.84) (Table S2). Conversely, miR‐30d‐5p (log_2_FC = −1.11; Figure 1D) and miR‐937‐3p (log_2_FC = −2.23) resulted down‐regulated (Table S2). miR‐151a‐3p (Figure 1C) and miR‐30d‐5p (Figure 1D) showed similar significant expression levels also when also unmatched tumor tissue samples (n = 574) were included in the analysis.

#### 3.1.4 Analysis of RNA‐seq in tissue samples

**FIGURE 2:**
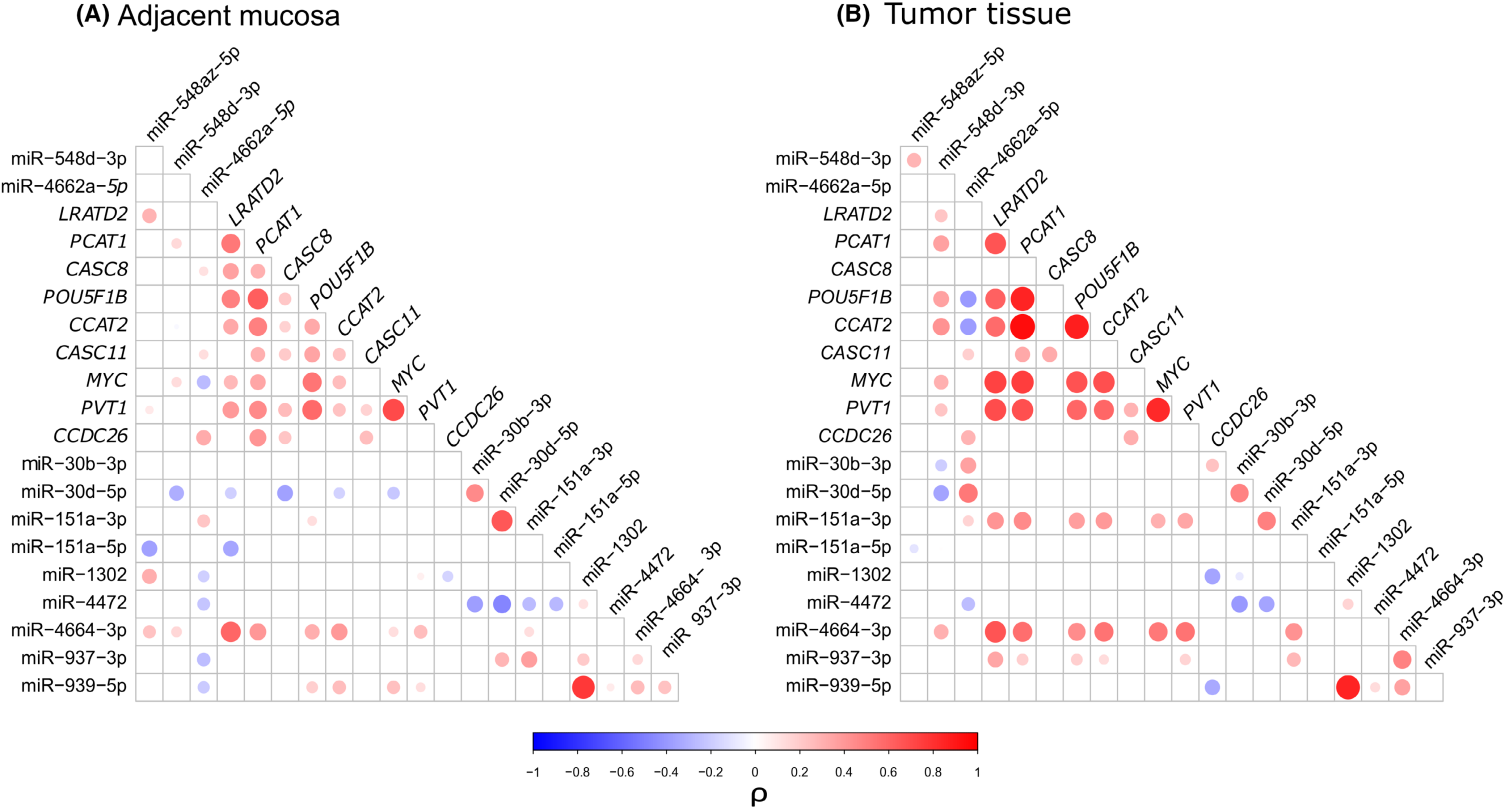
Spearman correlation analysis between expression levels of miRNAs and CRC‐related genes in the 8q24 region. Results are reported separately for (A) adjacent mucosa and (B) tumor tissue. The size of the dots is proportional to the significance of the correlation test. Only significant correlations (*p* ≤ 0.05) are reported. The color intensity represents the correlation coefficient (ρ; blue and red for negative and positive correlations, respectively).

From co‐expression analysis among DE miRNAs and CRC‐relevant genes from the literature residing in the 8q24 region (*MYC*, *PVT1*, *LRATD2*, *POU5F1B*, *PCAT1*, *CCAT2*, *CASC8*, *CASC11*, and *CCDC26*), several significant correlations (*p* < 0.05) were observed either in adjacent mucosa (Figure 2A, Table S3A) or in tumor tissue (Figure 2B). Specifically, in tumor tissue, miR‐548d‐3p, miR‐151a‐3p, and miR‐937‐3p showed significant positive correlations (0.18 < ρ < 0.41, Table S3B) with *LRATD2*, *PCAT1*, *POU5F1B*, *CCAT2*, *PVT1*, and *MYC* (except for miR‐937‐3p) and this pattern was not observed in adjacent mucosa. miR‐4664‐3p was correlated with these genes both in tumor and adjacent mucosa. Additionally, in CRC tissue, miR‐1302 and miR‐939‐5p were negatively correlated with *CCDC26* (ρ = −0.35 and ρ = −0.34, respectively).

miR‐151a‐5p and miR‐30d‐5p showed negative correlations especially in adjacent mucosa: miR‐30d‐5p with *LRATD2* (ρ = −0.19); *CASC8* (ρ = −0.36); *CCAT2* (ρ = −0.18) and *MYC* (ρ = −0.19); miR‐151a‐5p with *LRATD2* (ρ = −0.35).

Co‐expression was also analyzed between miRNAs to highlight any distinctive pattern. Several correlations (*n* = 24 in adjacent mucosa and *n* = 21 in tumor tissue) were identified between miRNA expression levels, especially among those belonging to the same cluster, as miR‐30b‐3p and miR‐30d‐5p (ρ = −0.49). Interestingly, also miRNAs coded by genomically distant genes, as miR‐1302 and miR‐939‐5p (distance ~3 Mb) showed a high correlation both in adjacent (ρ = −0.77) and CRC tissue (ρ = −0.85) (Figure 2).

#### 3.1.5 *MYC* amplification

To exclude the possibility that the observed up‐regulation of miRNAs could be driven by the amplification of the 8q24 region as previously observed in other tumor types, a DNA variant analysis was performed on the subset of CRC patients with the TSO500‐HT data available.

TSO500‐HT data were analyzed to assess the presence of gene amplification in the 8q24 region, especially for *MYC* and the surrounding loci (Table S4A). The panel includes *MYC*, *RAD21*, and *RECQL4* among the genes annotated in the 8q24 region. However, only *MYC* is evaluated for its copy‐number variation levels in the TSO500 HT analysis pipeline. Out of 99 CRC patients with available data, 29 individuals (29.3%) showed at least one gene amplification. However, only nine CRC patients (9.1%) carried amplifications of *MYC*. Analysis of TCGA COAD and READ data showed an amplification of *MYC* in 41 samples out of 616 patients (6.66%, Figure S2A). The proportion of CRC patients with *MYC* amplification was similar between our study and the data reported in TCGA COAD and READ datasets, while the number of patients carrying an amplification of the same gene was higher for other type of cancer (such as ovarian cancer, 41%; breast cancer, 21.2%; small cell lung cancer, 10.38%; Figure S2A). Finally, a new differential expression analysis with the exclusion of the samples (both adjacent mucosa and tumor tissues) of patients with a *MYC* amplification was carried out. Ten miRNAs (miR‐1302, miR‐151a‐3p, miR‐30b‐3p, miR‐30d‐5p, miR‐4662a‐5p, miR‐4664‐3p, miR‐4664‐5p, miR‐548az‐5p, miR‐548d‐3p and miR‐937‐3p) resulted differentially expressed after the exclusion of *MYC*‐amplified samples (Table S4B).

#### 3.1.6 Functional enrichment analysis

In total, 213 genes are targeted by miRNAs up‐regulated in tumor tissues, as retrieved from the miRWalk database (Table S5A). miR‐4472 shared one target (*ATPAF1*) with miR‐151a‐5p, four targets (*B4GALT5*, *MSN*, *PSAP* and *TMEM184B*) with miR‐939‐5p and *CASTOR2* with miR‐4664‐3p. miR548az‐3p shared *SECISBPL2* with miR‐939‐5p (Figure S2). The functional analysis based on the above 213 target genes (Table S5A) highlighted that among the top 20 enriched clusters of terms, the most significant were “translational elongation” (GO: GO:0006414, adj *p*‐value = 0.027), “TP53 Regulates Transcription of Genes Involved in G2 Cell Cycle Arrest” (R‐HSA‐6804114, adj *p*‐value = 0.026), and “cell cycle” (R‐HSA‐1640170, adj *p*‐value = 0.026), (Table S5B).

The down‐regulated miRNAs target in total 420 genes (Table S6A). miR‐30b‐3p and miR‐30d‐5p share one target (*MRO*), while miR‐30b‐3p and miR‐4662a‐5p two (*GLP2R and ZNF70*) (Figure S3). The top 20 enriched clusters of terms included “endomembrane system organization” (GO:0010256, adj *p*‐value < 0.0001), “proteasomal protein catabolic process” (GO:0010498 adj *p*‐value = 0.005), “negative regulation of protein phosphorylation” (GO:0001933, adj *p*‐value = 0.005) and “positive regulation of cellular catabolic process” (GO:0031331, adj *p*‐value = 0.005) (Table S6B).

#### 3.1.7 Small RNA sequencing in surrogate tissues

##### Stool samples

Out of the 54 miRNAs detected in the 8q24 region, four were differentially expressed in fecal samples of CRC cases in comparison with controls. Specifically, in patients, miR‐6849‐5p was down‐regulated (log_2_FC = −0.71, adj *p*‐value = 0.05; Table S7A) while miR‐151a‐3p (log_2_FC = 1.40, adj *p*‐value < 0.0001; Figure 1E), miR‐30d‐5p (log_2_FC = 4.10, adj *p*‐value < 0.0001; Figure 1F), and miR‐10400‐5p (log_2_FC = 2.46 adj *p*‐value < 0.0001) were up‐regulated (Table S7A). After tumor stratification by disease stage, miR‐10400‐5p and miR‐30d‐5p, resulted up‐regulated compared to healthy controls, in both stage I–II (log_2_FC = 1.93 and log_2_FC = 3.13, respectively) and stage III–IV (log_2_FC = 2.62; log_2_FC = 5.23) with an increasing trend. miR‐6849 was down‐regulated in CRC stage I–II (log_2_FC = −1.44) and stage III–IV (log_2_FC = −1.04) when compared to healthy controls. miR‐151a‐3p (log2FC = 2.08) and miR‐1302 (log2FC = −0.97) were, respectively, up‐ and down‐regulated only in stage III‐IV (Table S7B).

By stratifying CRC patients by the tumor grade, miR‐30d‐5p, miR‐151a‐3p, and miR‐10400‐5p were up‐regulated in both G1‐G2 and G3 compared to controls, with miR‐10400‐5p following an increasing trend (log_2_FC = 2.94, adj *p*‐value < 0.0001). In G3 patients, also miR‐1302 (log_2_FC = −0.74, adj *p*‐value = 0.04) and miR‐6849‐5p (log_2_FC = −1.04, adj *p*‐value = 0.01) were differentially expressed (Table S7C).

Finally, after a stratification of CRC cases for tumor localization, miR‐30d‐5p was up‐regulated both in colon/sigma (log_2_FC = 4.11, adj *p*‐value < 0.0001) and rectal cancer (log_2_FC = 4.72, adj *p*‐value < 0.0001) when compared to healthy controls while miR‐6849‐5p was down‐regulated in colon/sigma patients only (log_2_FC = −1.00, adj *p*‐value = 0.01) (Table S7D).

##### Plasma EVs samples

No miRNAs in the 8q24 region resulted differentially expressed in plasma EVs from CRC samples when compared to controls (Table S8A–D), with the only exception represented by miR‐30d‐5p, significantly down‐regulated in samples from patients with G1‐G2 tumors (log2FC = −0.31, adj. *p*‐value = 0.02) (Table S8C).

### 3.2 Study II

#### 3.2.1 Study population

Urine samples were collected from all 116 individuals of the Study II cohort (66 BC cases and 50 controls, all men) while plasma EVs samples were available for 93 of them. The mean age recorded for cases was 64.2 ± 6.9 years and for the control group was 64.6 ± 7.4 years (Table 1). In both plasma and urine samples of this population, 43 out of the 54 region‐specific miRNAs were detected.

#### 3.2.2 Small RNA sequencing in BC tissue from TCGA

**FIGURE 3:**
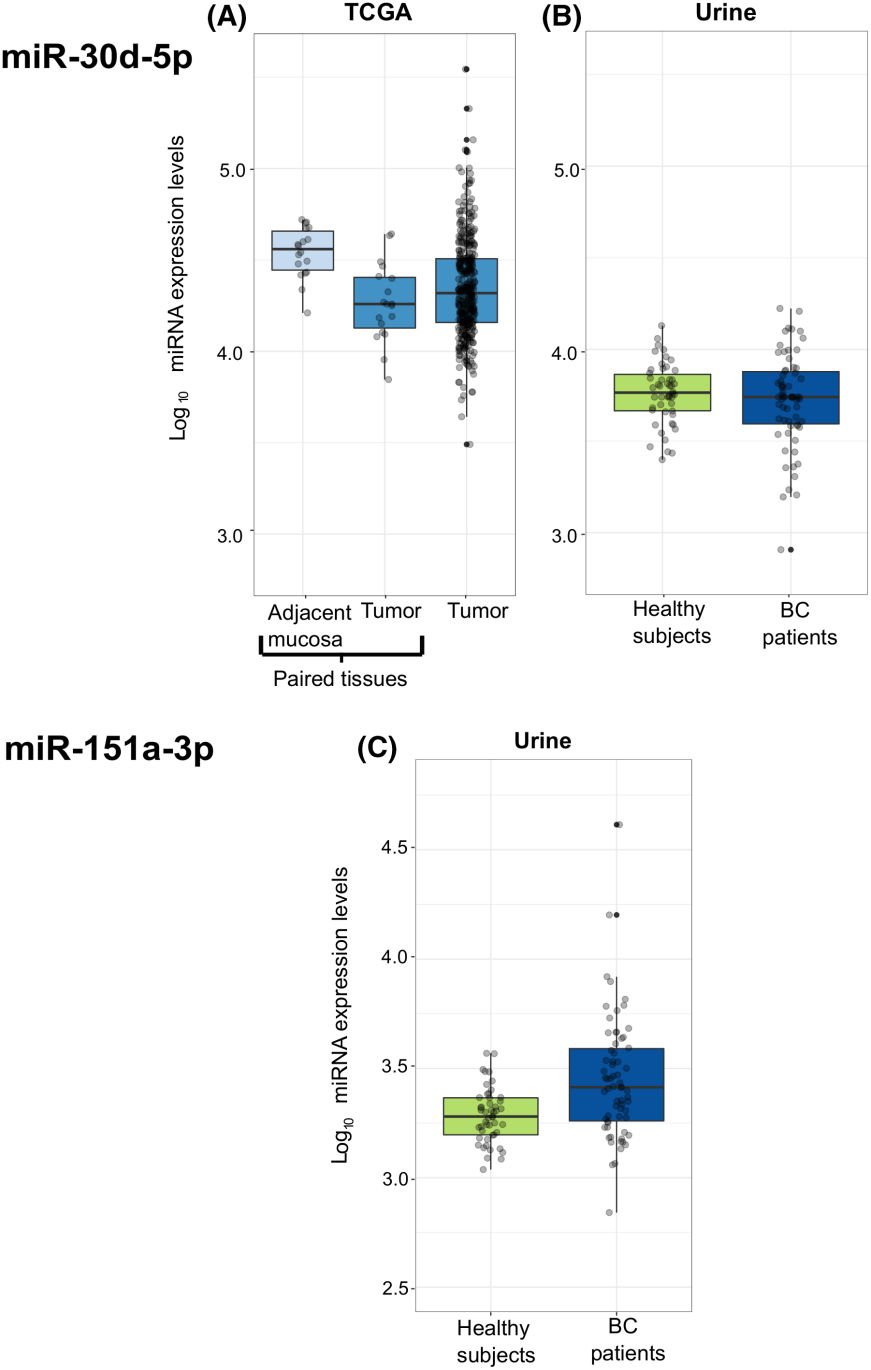
Box plots representing expression levels of miR‐30d‐5p as measured in (A) TCGA‐BLCA database (both for paired and not paired BC tissues) and (B) urine samples from BC patients and healthy controls of Study II. (C) Box plots representing expression levels of miR‐151a‐3p as measured in urine samples from BC patients and healthy controls of Study II.

No tissue samples were available for this study cohort; therefore, the TCGA repository was explored to evaluate 8q24 miRNA expression in BC tissues (all samples being MIBC). Five miRNAs (miR‐151a‐5p, miR‐30b‐5p, miR‐30d‐5p [Figure 3A], miR‐4666a‐5p, miR‐937‐3p) were differentially expressed when comparing BC (*n* = 19) to adjacent mucosa (*n* = 19) (Table S9). Four of them were also dysregulated in CRC tissues with miR‐151a‐5p, miR‐30d‐5p and miR‐937‐3p showing the same trend of expression. On the other hand, miR‐30b‐5p, which was down‐regulated in BC, was altered in the CRC tissues of TCGA but in an opposite way.

##### Urine

Testing the differences between BC cases and controls in urine samples, three miRNAs resulted differentially expressed (all with adj *p*‐value < 0.05): miR‐30b‐5p (log2FC = −0.85), miR‐30d‐5p (log2FC = −0.32; Figure 3B), and miR‐151a‐3p (log2FC = 0.481; Figure 3C) (Table S10).

##### Plasma EVs samples

No significant differentially expressed miRNAs were identified in plasma EVs from BC patients and controls. (Table S11).

#### 3.2.3 Co‐expression analysis

Seven genes (*ATPAF1*, *SECISBP2L*, *MSN*, *B4GALT5*, *PSAP*, *TMEM184B* and *GATSL2*) were annotated as validated targets of at least two CRC up‐regulated miRNAs. For them, miRNA‐target co‐expression analysis was evaluated in the TCGA‐COAD and READ datasets and seven significant (p < 0.05) anti‐correlations were identified (Table S12A). Considering the down‐regulated miRNAs, the co‐expression between three validated gene targets (*GLP2R*, *MRO* and *ZNF70*) and three miRNAs (miR‐30b‐3p, miR‐30d‐5p and miR‐4662a‐5p) was investigated and two significant anti‐correlations were found (Table S12A).

The same analyses were performed on the TCGA‐BLCA dataset, and 12 significant (*p* < 0.05) anti‐correlations were identified between the up‐regulated miRNAs and their targets. Two anti‐correlations were found for the down‐regulated miRNAs. Five interactions (miR‐151a‐5p—*Moesin [MSN]*; miR‐151a‐5p—*PSAP*; miR‐4664‐3p—*PSAP*; miR‐548d‐3p—*SECISBP2L*, and miR‐548d‐3p—*PSAP*) overlapped with the ones obtained in the TCGA‐CO‐READ analysis. Similarly, one interaction (miR‐30b‐3p—*MRO*) was detected in common between the down‐regulated miRNAs in TCGA‐CO‐READ and TCGA‐BLCA datasets (Table S12B).

## 4 DISCUSSION

In this study, we aimed at investigating epigenetic alterations, namely miRNA expression dysregulation, as a less explored aspect in the still unexplained relationship between the 8q24 region and the onset of several malignancies. Therefore, we comprehensively analyzed the expression profiles of all miRNA genes residing in the locus by next‐generation sequencing in multiple biospecimens from a group of CRC patients and healthy controls (tumor tissue and paired adjacent mucosa for patients, stool, and plasma EVs for both). To assess if the dysregulation of these miRNAs was specific of CRC or a more generalized alteration in solid tumors, we explored their profiles in BC tissues from TCGA, as well as in urine and plasma EVs samples from BC patients and healthy controls collected in our previous studies.^19^, ^22^

In CRC tissues, out of the 54 identified mature miRNAs in the 8q24 region, 12 were differentially expressed between tumor and adjacent mucosa, with nine of them being up‐regulated. Some of the latter miRNAs have been previously reported as over‐expressed also in other cancers tissues. For example, miR‐4472 has been found up‐regulated in highly metastatic breast tissue,^32^ as well as miR‐939‐5p in pancreatic cancer tissue when compared to adjacent mucosa.^33^ On the other hand, two of the three down‐regulated miRNAs, miR‐30b‐3p and miR‐30d‐5p, belong to the same cluster and for both their reduced expression levels have been described before in several different cancers.^34^, ^35^, ^36^, ^37^, ^38^, ^39^

After the stratification by tumor stage, we observed a decreasing trend of expression for miR‐30d‐5p going from adjacent mucosa to stage III–IV tumors, while miR‐937‐3p and miR‐939‐5p exhibited an increasing trend. For miR‐939‐5p a similar up‐regulation was also observed in pancreatic cancer, where miRNA expression levels increased in advanced tumor stages.^33^ According to tumor grade, miR‐30d‐5p and miR‐4662a‐5p showed a decreased expression from adjacent mucosa to G3 tumor, while miR‐548d‐3p and miR‐937‐3p had an increased expression trend. This has never been observed before. Interestingly, similar results were confirmed in the TCGA‐CRC dataset with five out of 12 miRNAs (miR‐151a‐3p, miR‐151a‐5p, miR‐30b‐5p, miR‐4662a‐5p, miR‐30d‐5p) dysregulated in the same direction as in our dataset.

We explored the correlations among the altered miRNAs in tissues and CRC‐related genes located in 8q24. In CRC tissue, miR‐151a‐3p, miR‐4664‐3p, and miR‐937‐3p were all positively related with the expression levels of several genes, including *MYC* and *PVT1*. This correlation was higher than in adjacent mucosa, thus suggesting a possible common regulatory mechanism driving the observed overexpression. For example, 8q24 region harbors several clusters of enhancers involved in long‐range chromatin interactions.^40^ Despite experimental validations are needed, genes might be involved in such regulatory interactions resulting in a combined transcriptional alteration among distal genomic loci. We also found correlations among the altered miRNAs: for example, miR‐4472 showed a negative correlation with miR‐30d‐5p and miR‐30b‐5p in both adjacent mucosa and in tumor tissue with all sharing some target genes involved in apoptotic processes (data not shown). Surprisingly, miR‐30d‐5p and miR‐151a‐3p, despite their opposite trend in the comparison between adjacent mucosa and tumor tissue, resulted positively correlated among them. Interestingly, the negative correlation observed between miR‐30d‐5p and several CRC‐relevant genes could be explained by a possible endogenous miRNA sponge mechanism mediated by lncRNAs that could act as inhibitor of miRNA function. For instance, Yu et al. observed that *PVT1*, a lncRNA upstream of miR‐30d‐5p gene, resulted to work as a competing endogenous RNA in CRC through the PVT1/miR‐30d‐5p/RUNX2 axis.^41^, ^42^ In this case, *PVT1* was able to directly interact with miR‐30d‐5p, resulting in a competitive binding that decreased miR‐30d‐5p abundance and relieved the repression mediated by this miRNA on the downstream target *RUNX2*, an oncogene related to tumor growth and metastasis.

The possible mechanism involved in the miRNA dysregulation is still not well understood. For the up‐regulated miRNAs, their expression may be altered by the amplification of loci in the region. The observed up‐regulation of the 8q24‐related miRNAs could be an indirect consequence of the amplification of *MYC*, one of the most common copy‐number alteration on chromosome 8.^43^, ^44^ However, the genomic profiling of CRC patients in Study I showed that only a small fraction of them harbored a *MYC* amplification, and this proportion was confirmed by the data from the TCGA CO‐READ dataset. Moreover, excluding *MYC*‐amplified tumors from the differential expression analysis, confirmed the altered pattern of expression observed with the complete cohort. We may therefore hypothesize that the dysregulation of the observed miRNAs could be due to alternative underlying mechanisms other than amplification, such as epigenetic effects (including methylation or histone modification^45^) or the presence of genetic variants that could work as expression quantitative loci for miRNAs.^46^, ^47^

To further investigate the possible targets of the differentially expressed miRNAs in CRC tissues, we retrieved all their experimentally validated targets from miRWalk database. The functional analysis of these target genes highlighted important enriched clusters involved in cell cycle related genes regulated by TP53. This is consistent with the aberrant up‐regulation of these miRNAs, which may target genes belonging to tumor‐suppressive signaling pathways.

miRNA levels were then investigated in fecal samples to understand if a surrogate biospecimen could mirror the signals obtained in tissue samples and in EVs. In this respect, miR‐30d‐5p and miR‐151a‐3p also resulted dysregulated in stool of CRC patients with respect to healthy controls, with miR‐30d‐5p showing an increasing trend of expression going from healthy controls to patients with high disease stage. This apparent contradiction with results from tissues could be explained by the expression levels of miR‐30d‐5p that are higher in both CRC patient stool and tissues, regardless of a decreasing trend from adjacent mucosa to tumor tissue, in comparison with stool of healthy controls (as shown in Figure 3). We speculate that this miRNA could be over‐expressed in stool in presence of a malignancy but this up‐regulation could be driven by the microenvironment surrounding the tumor influencing also the adjacent mucosa. Bjørnetrø et al^39^ found indeed that hypoxic colonic cancer cells release vesicles containing miR‐30d‐5p and thus contribute to its high levels in adjacent mucosa.

These observations allowed us to hypothesize that the dysregulation of certain miRNAs in the 8q24 regions is a reflection of the presence of a cancer and that the dysregulation could be measured also in surrogate tissues in direct contact with the tumor (such as stool) but not in peripheral surrogate liquid biopsy like plasma EVs.

Finally, to assess if the observed miRNA alterations in CRC are detectable also in other cancers, we explored their levels in TCGA BCLA dataset and five miRNAs resulted also dysregulated in this tumor. Interestingly, four of them were altered both in CRC and BC tissues with the same trend of expression, while only one had an opposite direction. As for CRC, we investigated the expression of these miRNAs in the urine as surrogate tissue for BC and identified three miRNAs, miR‐151a‐3p, miR‐30d‐5p, and miR‐30b‐5p, differentially expressed. Both miR‐30d‐5p and miR‐30b‐5p had the same trend of expression as in tissues.

Notably, no miRNA in the 8q24 region resulted differentially expressed in plasma EVs from both CRC and BC samples when compared to controls. This may be partially explained by the large majority of non‐metastatic and not‐invasive tumors included in the study.

Co‐expression analyses of dysregulated miRNAs and their target genes highlighted an interesting interaction between *MSN* and both miR‐151a‐5p and miR‐548d‐3p. MSN is a key component of the *Ezrin*/*Radixin*/*Moesin* (ERM) complex, which crosslinks several proteins to the actin filaments of cytoskeleton. Kobori et al.,^48^ investigated the role of ERM and MSN in the expression of PD‐L1 and found, in CRC cell lines, that the silencing of *MSN* lead to an over‐expression of PD‐L1 and a consequent poor diagnosis. In our data, *MSN* seems to be down‐regulated by miR‐151a‐5p and to a lesser extent by miR‐548d‐3p and these findings could partially explain this mechanism. However, considering that miRNAs are post‐transcriptional regulators of gene expression, the correlations of RNA levels may not be reflecting a direct effect of miRNAs on the protein levels of their targets. Further experimental validations in a controlled system are needed to better characterize these molecular interactions.

In summary, several dysregulated miRNAs mapping to chromosome 8q24 were found in CRC and BC, both in primary and surrogate tissues. The altered miRNAs emerged from an investigation of the whole miRNome, highlighting the importance of the 8q24 locus also at the transcriptomic level. The strength of this study is that we took advantage of a large collection of several biospecimens from the same patients. Tissues, stool, and plasma samples were available for many CRC patients. In this respect, no previous study assessed 8q24 miRNA profiles in stool of CRC patients and healthy controls.

The present study has emphasized the importance of investigating regulatory RNA molecules in this genetic desert associated with cancer. Additional studies are required to assess the possible functions and their role in tumorigenesis of the dysregulated miRNAs identified, as well as to expand the investigation also to other cancer types.

## AUTHOR CONTRIBUTIONS

**Amedeo Gagliardi:** Data curation (lead); formal analysis (lead); investigation (supporting); methodology (equal); validation (equal); visualization (equal); writing – original draft (lead); writing – review and editing (lead). **Giulia Francescato:** Data curation (lead); formal analysis (equal); investigation (lead); methodology (lead); validation (equal); visualization (equal); writing – original draft (lead); writing – review and editing (lead). **Giulio Ferrero:** Conceptualization (equal); data curation (equal); formal analysis (equal); methodology (equal); supervision (supporting); visualization (equal); writing – original draft (equal); writing – review and editing (equal). **Giovanni Birolo:** Data curation (equal); formal analysis (equal); writing – original draft (equal); writing – review and editing (equal). **Sonia Tarallo:** Conceptualization (equal); investigation (equal); methodology (equal); writing – original draft (equal); writing – review and editing (equal). **Antonio Francavilla:** Data curation (equal); investigation (equal); writing – original draft (equal); writing – review and editing (equal). **Giulia Piaggeschi:** Investigation (equal); methodology (equal); writing – original draft (equal); writing – review and editing (equal). **Carla Di Battista:** Investigation (equal); methodology (equal); visualization (equal); writing – original draft (equal); writing – review and editing (equal). **Gaetano Gallo:** Data curation (equal); writing – original draft (equal); writing – review and editing (equal). **Alberto Realis Luc:** Data curation (lead); writing – original draft (equal); writing – review and editing (equal). **Carlotta Sacerdote:** Data curation (lead); writing – original draft (equal); writing – review and editing (equal). **Giuseppe Matullo:** Data curation (equal); supervision (equal); writing – original draft (equal); writing – review and editing (equal). **Paulo Vineis:** Conceptualization (equal); supervision (equal); writing – original draft (equal); writing – review and editing (equal). **Alessio Naccarati:** Conceptualization (lead); data curation (equal); funding acquisition (lead); investigation (equal); methodology (equal); supervision (lead); visualization (supporting); writing – original draft (lead); writing – review and editing (lead). **Barbara Pardini:** Conceptualization (lead); data curation (supporting); funding acquisition (equal); investigation (equal); methodology (equal); project administration (lead); supervision (equal); visualization (equal); writing – original draft (lead); writing – review and editing (lead).

## CONFLICT OF INTEREST

None declare.

## Supporting information


Figure S1

Figure S2

Figure S3

Table S1

Table S2

Table S3

Table S4

Table S5

Table S6

Table S7

Table S8

Table S9

Table S10

Table S11

Table S12

## Data Availability

All the data relevant of the study are included in the article or uploaded as supplementary information. Raw data access is available upon request to the corresponding author.
