# Supplementary figures and images for: The 8q24 region hosts miRNAs altered in biospecimens of colorectal and bladder cancer patients
Source: Cancer Med. 2022 Nov 10;12(5):5859–73. doi: 10.1002/cam4.5375 (PMC10028171; doi:10.1002/cam4.5375)

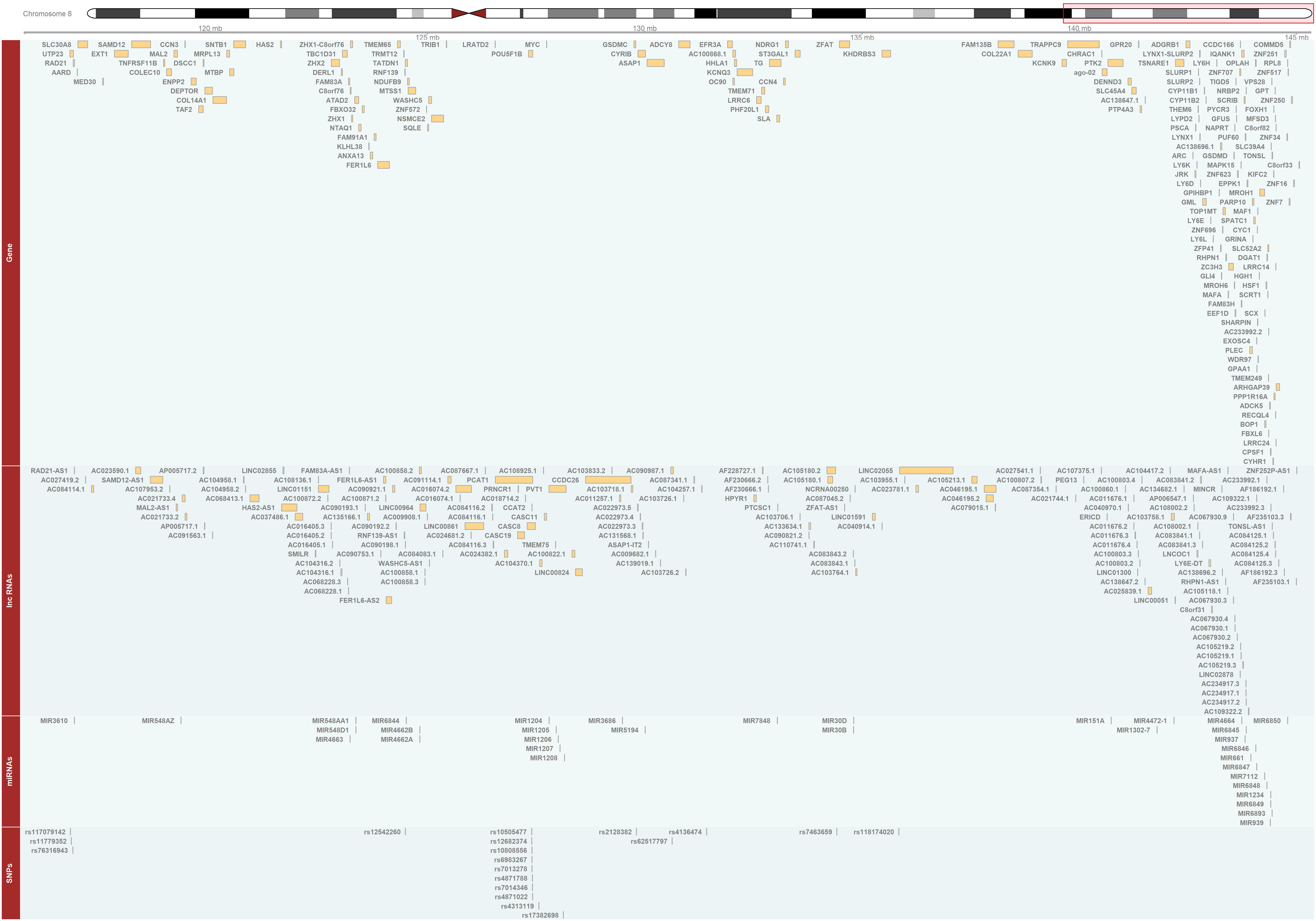

Supplement: Supplementary file 1 — Figure S1 [file CAM4-12-5859-s002.jpg]

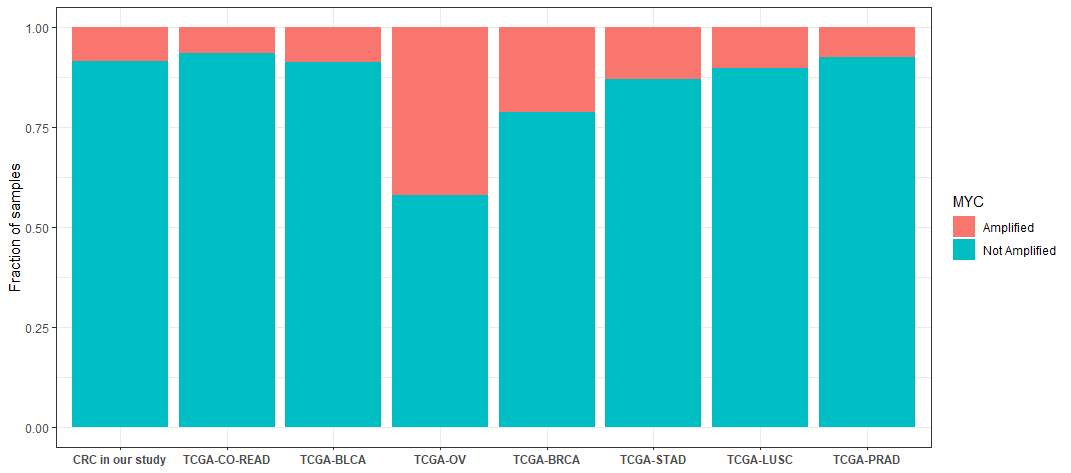

Supplement: Supplementary file 2 — Figure S2 [file CAM4-12-5859-s015.tiff]

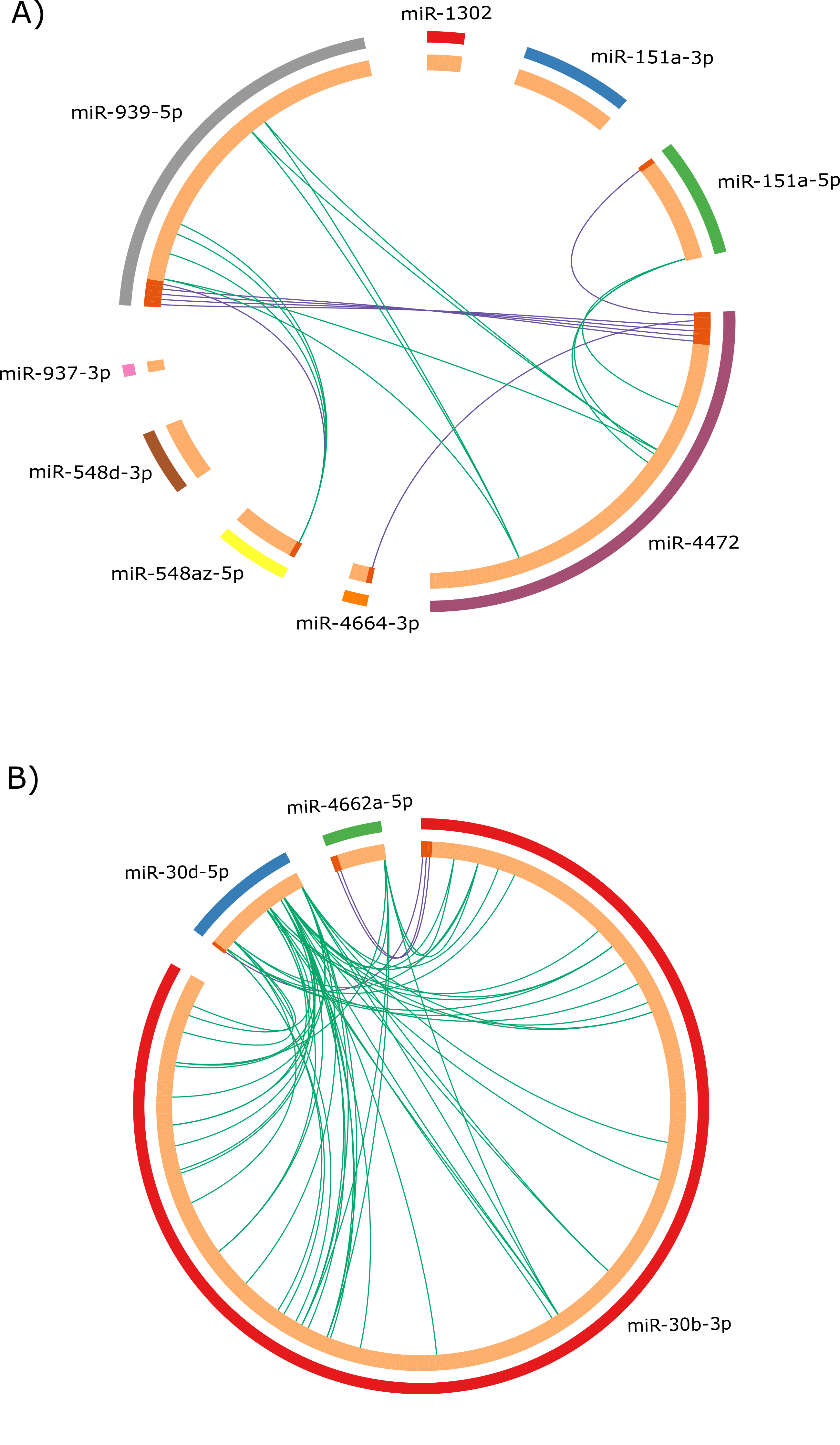

Supplement: Supplementary file 3 — Figure S3 [file CAM4-12-5859-s007.tiff]
